# Long-Term Outcome of Healthy Participants with Atrial Premature Complex: A 15-Year Follow-Up of the NIPPON DATA 90 Cohort

**DOI:** 10.1371/journal.pone.0080853

**Published:** 2013-11-19

**Authors:** Taku Inohara, Shun Kohsaka, Tomonori Okamura, Makoto Watanabe, Yasuyuki Nakamura, Aya Higashiyama, Aya Kadota, Nagako Okuda, Takayoshi Ohkubo, Katsuyuki Miura, Akira Okayama, Hirotsugu Ueshima, for the NIPPON DATA 80/90 Research Group

**Affiliations:** 1 Department of Cardiology, Keio University School of Medicine, Tokyo, Japan; 2 Department of Preventive Medicine and Public Health, Keio University School of Medicine, Tokyo, Japan; 3 Department of Preventive Cardiology, National Cerebral and Cardiovascular Center, Suita, Japan; 4 Department of Cardiovascular Epidemiology, Kyoto Women’s University, Kyoto, Japan; 5 Department of Environmental and Preventive Medicine, Hyogo College of Medicine, Nishinomiya, Japan; 6 Department of School Nursing and Health Education, Osaka Kyoiku University, Kashiwara, Japan; 7 Department of Nutritional Epidemiology, National Institute of Health and Nutrition, Tokyo, Japan; 8 Department of Hygiene and Public Health, Teikyo University School of Medicine, Tokyo, Japan; 9 Department of Health Science and Center for Epidemiologic Research in Asia; 10 First Institute for Health Promotion and Health Care, JATA, Tokyo, Japan; University of Louisville, United States of America

## Abstract

**Background:**

Atrial premature complexes (APC) are among the most frequently encountered electrocardiographic abnormalities. However, their prognostic value among healthy individuals is unclear. This study aimed to clarify the role of APC in predicting cardiovascular events in a large Japanese community cohort using long-term follow-up data.

**Methods:**

National Integrated Project for Prospective Observation of Non-communicable Disease And its Trends in the Aged, 1990-2005, (NIPPON DATA 90) was a large cohort study of cardiovascular disease (CVD) in Japan. A total of 7692 otherwise healthy participants with no history of myocardial infarction, stroke, atrial fibrillation, or atrial flutter were enrolled (men, 41.5%; mean age, 52.5 ± 13.7 years).

**Results:**

A total of 64 (0.8%) participants had at least one beat of APC on screening 12-lead electrocardiogram. During the follow-up of 14.0 ± 2.9 years (total, 107,474 patient-years), 338 deaths occurred due to CVD. The association between APC and CVD outcome was assessed using Cox proportional hazard models. Cox regression analysis revealed that the presence of APC was an independent predictor for CVD deaths (HR: 2.03, 95% CI: 1.12–3.66, *P* = 0.019). The association of APC on CVD death was more evident in participants with hypertension (*P*-value for interaction, 0.03).

**Conclusions:**

APC recorded during the screening electrocardiogram are significantly associated with an increased risk of CVD deaths in a Japanese community-dwelling population and are a strong prognostic factor for hypertensive participants.

## Introduction

Cardiovascular disease (CVD) is the largest single cause of death, accounting for approximately 30% of all deaths worldwide[1]. Electrocardiographic (ECG) screening has been performed in various situations to identify the early signs of CVD, and atrial premature complexes (APC) are among the most frequently recorded abnormal findings[2,3]. Nevertheless, APC detected in participants with apparently normal hearts have been regarded as a relatively benign phenomenon and do not contribute to prognostic risk stratification.

 However, with the elaboration of cohort studies and large-scale registries in recent years, the potential hazards of premature complexes associated with various cardiac conditions have gradually been recognized. Frequent APC have been shown to be associated with a risk of atrial fibrillation, which subsequently leads to an increased risk of cerebral infarction[4-6]. Previous studies were limited in terms of being mostly derived from studies that used a 24-hour ambulatory ECG to evaluate relatively high-risk populations such as the elderly or those with certain risk factors, including hypertension. Furthermore, although atrial fibrillation leading to cerebral infarction is a major clinical event, the association of APC and actual mortality remains unclear. 

In this study, we sought to investigate the prognostic value of APC recorded during the screening ECG in a large community cohort study that enabled analyses of long-term CVD outcomes in Japan.

## Methods

### Participants

The cohort studies of the National Survey on Circulatory Disorders, Japan, are referred to as NIPPON DATA (National Integrated Project for Prospective Observation of Non-communicable Disease And its Trends in the Aged), including a total of 2 cohort studies. The details of this cohort have been reported previously[7-19]. The baseline surveys were performed in 1980 and 1990 (NIPPON DATA 80 and NIPPON DATA 90). In the present study, we analyzed the data from NIPPON DATA 90, because the baseline survey of NIPPON DATA 80 recorded VPC and APC as a single category that was not further discriminated. 

A total of 8383 healthy participants (3504 men and 4879 women, ≥30 years of age) from 300 randomly selected districts throughout Japan were followed from 1990 until 2005. The total population aged ≥30 years in these selected districts was 10,956; therefore, the participation rate was 76.5% before exclusion for the reasons given below. The survey consisted of history taking, physical examinations, blood tests, standard 12-lead ECG recordings, and self-administered questionnaires on lifestyle.

**Figure 1:**
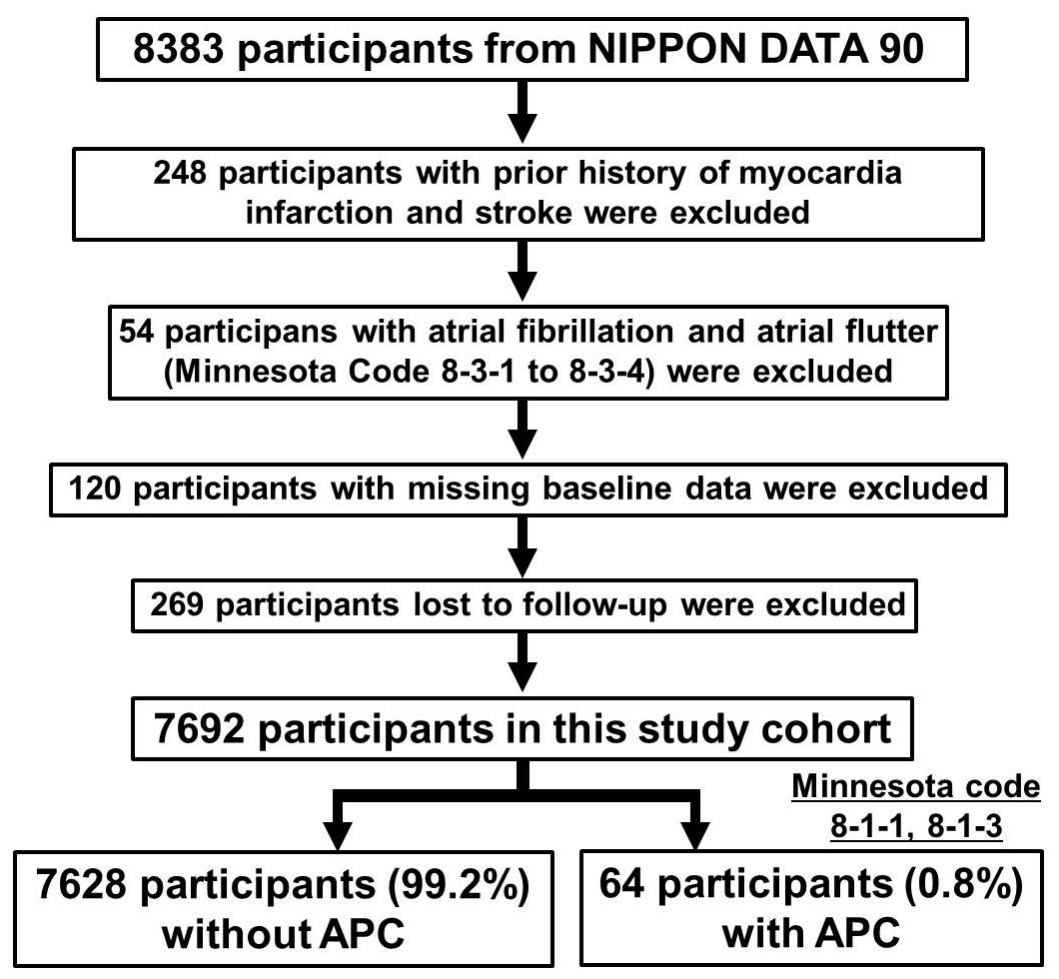
Study cohort creation. APC, atrial premature complexes.

 A total of 691 participants were excluded for the following reasons: history of a known vascular condition, such as myocardial infarction or stroke (n = 248); presence of atrial fibrillation or flutter (n = 54); some missing information at the baseline survey (n = 120); and absence of a permanent address that was needed to link to these vital statistical records (n = 269). The remaining 7692 otherwise healthy participants were included in our study (Figure 1).

### Baseline Examination

A standard 12-lead ECG was recorded in the resting supine position. Each ECG was reviewed independently by 2 trained coders according to the Minnesota Code (MC), as described previously[7-9,12,17]. Codes in agreement were accepted, whereas discordant results were adjudicated by a panel of study epidemiologists and cardiologists. Participants with at least one beat of APC (more than 10% of recorded QRS complexes) were determined as having APC; they were further classified according to the presence of APC (MC 8-1-1 or MC 8-1-3). Additional ECG findings examined included Q-wave abnormality (MC 1-1, 1-2, 1-3), QRS axis deviation (MC 2-1, 2-2, 2-3), high R wave (MC 3-1 to 3-4), ST depression (MC 4-1 to 4-4), T-wave abnormality (MC 5-1 to 5-5), intraventricular conduction block (MC 7-1-1 to 7-8), P-wave abnormality (MC 9-3-1 or 9-3-2), clockwise or counterclockwise rotation (MC 9-4-1 or 9-4-2), and ventricular premature complexes (MC 8-1-2 or MC 8-1-3).

Baseline blood pressures were measured by trained observers using a standard mercury sphygmomanometer on the right arm with the subject in a seated position. Hypertension was defined as systolic blood pressure of 140 mmHg or higher, diastolic blood pressure of 90 mmHg or higher, use of antihypertensive agents, or any combination of these. Non-fasting blood samples were centrifuged within 60 minutes of collection and were stored at -70°C until analysis. The blood samples were analyzed at the central laboratory (SRL, Tokyo, Japan) using established methods, as described previously[10,14-16]. Hypercholesterolemia was defined as serum total cholesterol ≥240 mg/dL, the use of medications for hypercholesterolemia, or both. Plasma glucose was also measured enzymatically. Diabetes mellitus was defined as plasma glucose ≥200 mg/dL, the use of medications for diabetes mellitus, or both. Body mass index was calculated as weight (kg) divided by the square of height (m). Public health nurses obtained information on smoking, drinking, and medical histories[13].

### Follow-up Survey

To determine the cause of death after the 15-year follow-up, we used the National Vital Statistics Database of Japan with permission from the Management and Coordination Agency, Government of Japan. The underlying causes of death were coded according to the International Classification of Diseases, Ninth Revision (ICD-9) until the end of 1994, and according to the International Classification of Diseases, Tenth Revision (ICD-10) from the beginning of 1995. The details of the follow-up survey and the classification of causes of death in the present study are described elsewhere[7-19]. Permission to use the National Vital Statistics was obtained from the Management and Coordination Agency, of the national government of Japan. Because of the voluntary participation of community dwellers to the national survey, of which data was matched to the National Vital Statistics, and anonymous nature of the data for the analysis, informed consent was waived. Approval for the study was obtained from the institutional review board of Shiga University of Medical Science (No. 12-18, 2000).

### Statistical Analysis

All data were expressed as mean ± standard deviation. Differences in each variable between participants with and those without APC were evaluated using the chi-square test or Fisher’s exact test for categorical variables and Student’s unpaired *t*-test for continuous variables. Event-free survival in patients with and those without APC was estimated by the Kaplan–Meier method, and statistical differences were evaluated by means of the log-rank test. Cox proportional hazard models were used to evaluate risk factor-adjusted associations of APC with each endpoint. 

In Cox models with endpoints including all-cause deaths and CVD deaths, adjustment was made in Models 1, 2, and 3. Covariates in Model 1 were age and gender. Model 2 consisted of Model 1 plus the conventional risk factors of body mass index, current smoking, drinking habit (never, past, and current drinker categories, with “never drinker” as a reference), hypercholesterolemia, diabetes mellitus, systolic blood pressure, and serum creatinine. Model 3 consisted of Model 2 plus other ECG findings (high R-wave, ST depression, T-wave abnormality, ventricular premature complexes). Covariates included in these models were clinically associated with CVD, significantly related to CVD deaths by univariate analysis, and also included the factors that were associated with APC at a level of significance *P* < 0.05 at baseline. 

Analyses of data were performed using SPSS, version 20 (SPSS Inc., Chicago, IL). All *P*-values were two-sided, and significance was defined as *P* < 0.05 for all analyses.

## Results

Sixty-four participants (0.8%) had APC, as determined by the screening ECG. The associated variables at baseline and the participants’ clinical outcomes are summarized in Table 1. Age, systolic blood pressure, and certain ECG findings (ST depression and T-wave abnormality) were significantly different between participants with and those without APC.

**Table 1:** Baseline characteristics of the study group.

	APC		
	No	Yes	
	(n = 7628)	(n = 64)	*p*
Male, n (%)	3159 (41.4%)	32 (50.0%)	0.165
Age, years	52.4	67.3	<0.001
BMI, kgm^-2^	22.9	22.0	0.022
Systolic blood pressure (mmHg)	135	148	<0.001
Diastolic blood pressure (mmHg)	81	83	0.111
Hypertension, n (%)	1606 (21.1%)	21 (32.8%)	0.022
Hypercholesterolemia, n (%)	517 (6.8%)	6 (9.4%)	0.411
Diabetes mellitus, n (%)	362 (4.8%)	4 (6.3%)	0.59
Current smoking, n (%)	2185 (28.6%)	16 (25.0%)	0.521
Drinking			
Ex-drinker	237 (3.1%)	2 (3.1%)	0.993
Current drinker	2156 (28.3%)	16 (25%)	0.564
Laboratory tests			
Total Cholesterol (mg/dL)	204	203	0.892
HDL Cholesterol (mg/dL)	54	53	0.741
Fasting blood glucose (mg/dL)	103	106	0.218
Creatinine (mg/dL)	0.81	0.85	0.119
Other ECG findings			
QRS axis deviation, n (%)	251 (3.3%)	2 (3.1%)	1
High R, n (%)	802 (10.5%)	12 (18.8%)	0.033
ST depression, n (%)	184 (2.4%)	6 (9.4%)	0.005
T-wave abnormality, n (%)	605 (7.9%)	15 (23.4%)	<0.001
Bundle branch block, n (%)	559 (7.3%)	4 (6.3%)	1
Rotation, n (%)	3810 (50%)	35 (54.7%)	0.45

Abbreviations: APC, atrial premature complex; BMI, body mass index; HDL, high density lipoprotein; ECG, electrocardiogram. All values are expressed as the mean ± SD or as a number with the percentage of participants in parentheses.

**Figure 2:**
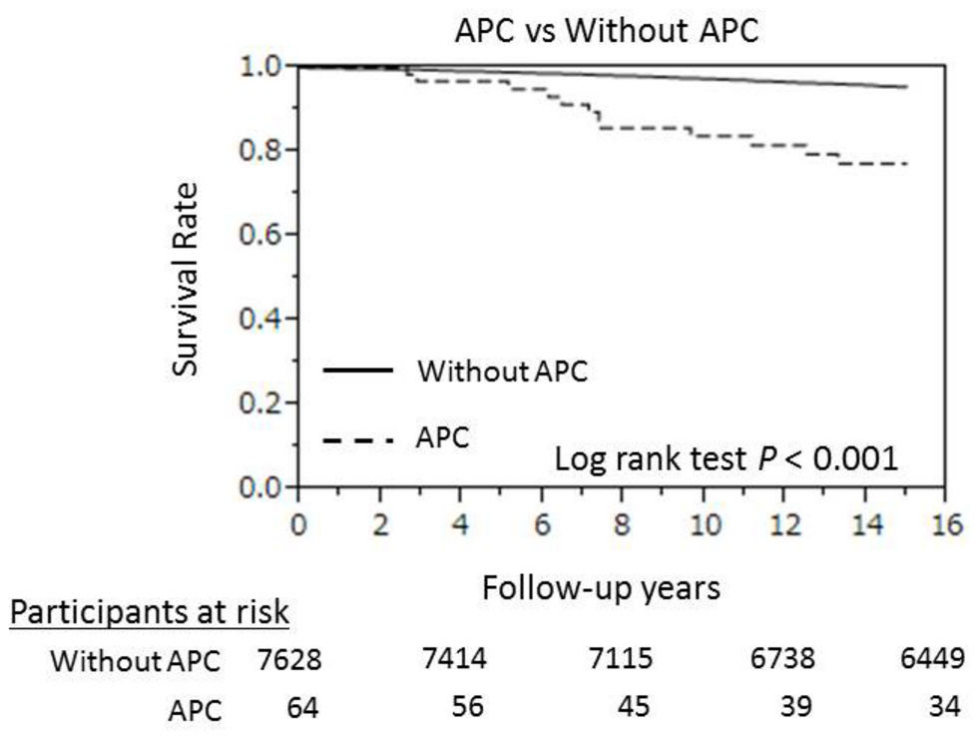
Kaplan–Meier estimates of cumulative hazard for cardiovascular disease for participants with and those without APC. APC, atrial premature complexes.

The total follow-up time for the 7692 participants (men, 41.5%; mean age, 52.5 ± 13.7 years) in this study was 107,474 person-years (mean, 14.0 ± 2.9 years). During the follow-up, there were 1211 all-cause deaths and 338 CVD deaths, including 68 due to coronary artery disease, 73 due to heart failure, and 138 due to stroke. Table 2 represents the cumulative mortality, which indicated that participants with APC tended to have higher all-cause, CVD, heart failure or stroke mortality. Figure 2 shows Kaplan-Meier survival curves for CVD deaths in participants with and without APC. These curves indicate that participants with APC at baseline had a significantly lower survival rate during the follow-up period (*P* < 0.001 by log-rank test). 

**Table 2:** Baseline ECG abnormalities and outcomes for the study group.

	APC		
	No	Yes	
	(n = 7628)	(n = 64)	*p*
Total death, n (%)	1181 (15.5%)	30 (46.9%)	<0.001
CVD death, n (%)	326 (4.3%)	12 (18.8%)	<0.001
CHD death, n (%)	67 (0.9%)	1 (1.6%)	0.388
HF death, n (%)	69 (0.9%)	4 (6.3%)	<0.001
Stroke death, n (%)	133 (1.7%)	5 (7.8%)	<0.001

Abbreviations: ECG, electrocardiogram; APC, atrial premature complex; CVD, cardiovascular disease; CHD, coronary heart disease; HF, heart failure. All values are expressed as a number with the percentage of participants in parentheses.

The statistical differences were evaluated using a log-rank test.

Cox regression analysis (Table 3) revealed that the presence of APC was an independent predictor for all-cause death (HR: 1.55, 95% CI: 1.07–2.24, *P* = 0.020) and CVD death (HR: 2.03, 95% CI: 1.12–3.66, *P* = 0.019).

**Table 3:** Hazard ratios for each outcome by ECG abnormality status at baseline.

	APC	*p* value
	HR (95% CI)	
All causes mortality		
Unadjusted	3.98 (2.77-5.71)	<0.001
Model 1	1.50 (1.04-2.16)	0.03
Model 2	1.60 (1.11-2.31)	0.012
Model 3	1.55 (1.07-2.24)	0.020
Cardiovascular death		
Unadjusted	5.79 (3.25-10.3)	<0.001
Model 1	2.06 (1.15-3.68)	0.015
Model 2	2.11 (1.17-3.81)	0.013
Model 3	2.03 (1.12-3.66)	0.019

Abbreviation: ECG, electrocardiogram; APC, atrial premature complex; HR, hazard ratio; CI, Confidence Interval.

Multivariate-Adjusted for Model 1 (age and gender), Model 2 (age, gender, body mass index, smoking habit, drinking habit, hypercholesterolemia, diabetes mellitus, systolic blood pressure, serum creatinine) and Model 3 (Model 2 + other ECG findings).

**Figure 3:**
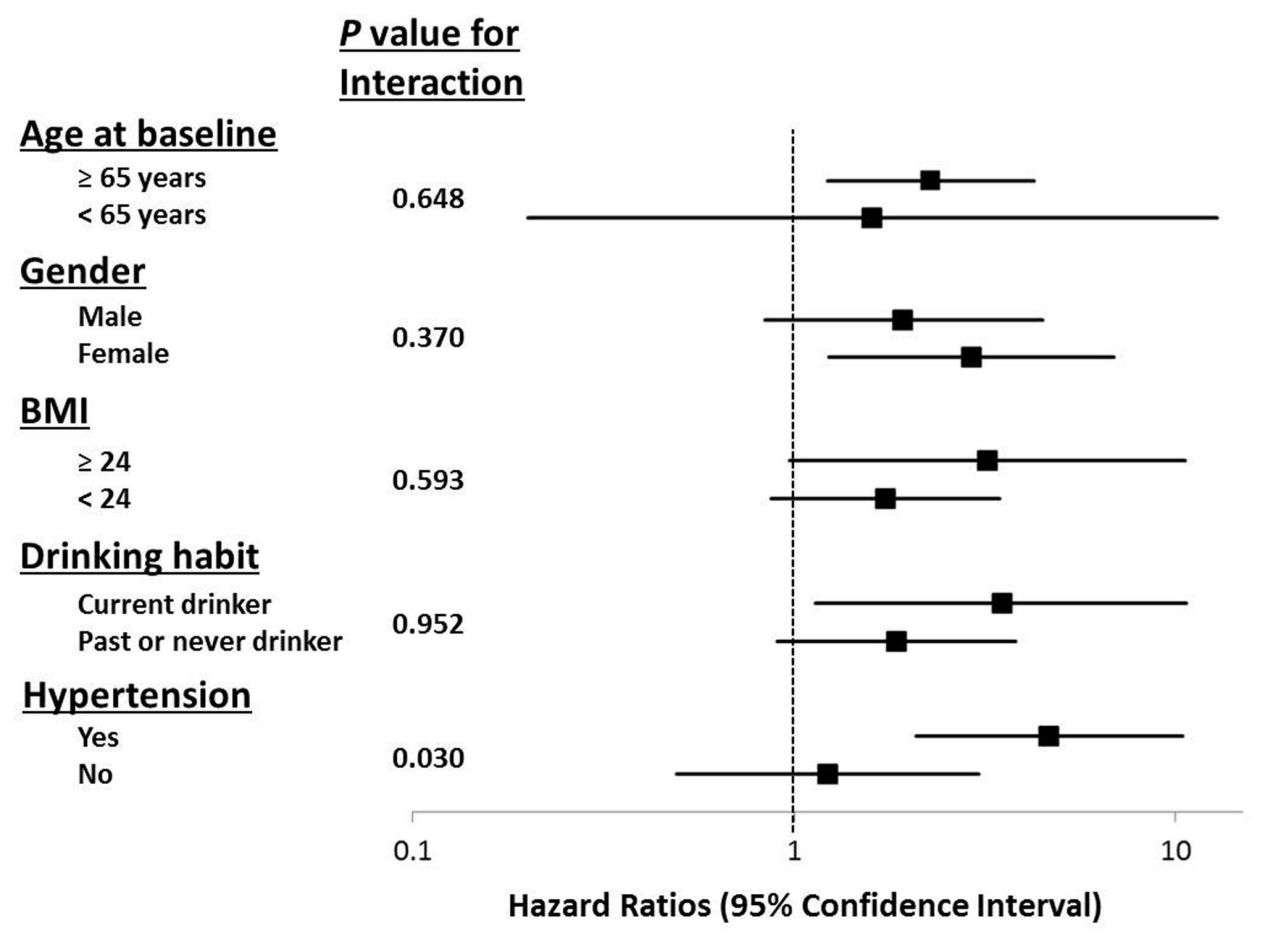
Adjusted hazard ratios of atrial premature complexes for cardiovascular disease deaths in various subgroups. Multivariate-adjusted Model 3 (age, gender, body mass index, smoking habit, drinking habit, hypercholesterolemia, diabetes mellitus, systolic blood pressure, serum creatinine, and other ECG findings). BMI, body mass index.

As shown in Figure 3, the impact of APC on CVD death was more evident in participants with hypertension. The probability value for an interaction between APC and hypertension on CVD death was 0.03. Indeed, the adjusted hazard ratios of APC for all-cause death (HR: 3.19, 95% CI: 1.87–5.45, *P* < 0.001) and CVD death (HR: 4.66, 95% CI: 2.09–10.4, *P* < 0.001) were statistically significant in the hypertensive participants. Further adjustment of anti-hypertensive medication did not alter the results (data not shown). Independent analysis of other major subgroups such as the elderly (≥65 years of age), women, obese individuals (body mass index ≥ 24 kg/m^2^) and current drinkers did not yield significant results (Figure 3).

## Discussion

The major finding of this prospective cohort study of a large sample of healthy Japanese individuals was that at least one beat of APC, recorded through the screening ECG, was associated with an increased risk of CVD mortality in healthy Japanese participants. Although APC was not frequently observed in our study (less than 1%), this long-term follow-up study clarified its potential hazards. Of note, this adverse effect of APC was particularly prominent in hypertensive participants. 

 The clinical significance of APC has been investigated for decades. Recent studies of the 24-hour ambulatory ECG have demonstrated that excessive APC are associated with a higher risk for the development of atrial fibrillation and CVD mortality[4,6,20]. Our findings extend the prognostic significance of APC to healthy participants who have premature complexes recorded on a routine 12-lead ECG. In this cohort, with a follow-up of more than 100,000 person-years (mean, 14.0 ± 2.9 years), the finding of APC on routine ECG was associated with an increase in CVD mortality, particularly due to heart failure and stroke. 

The mechanisms by which APC may increase the risk of heart failure are not clear, but several possibilities exist. First, APC may be an early manifestation of underlying heart disease that elevates left ventricular (LV) filling pressure. Early diastolic dysfunction may lead to elevated LV filling without clinical manifestations, with the exception of subtle left atrial remodeling that may be the cause of frequent APC[21]. A previous study conducted in the general community using Doppler echocardiography reported that although only approximately 5% of the population had systolic dysfunction, as many as approximately 30% had diastolic dysfunction, regardless of its severity, without any apparent heart failure[22]. In addition, some reports demonstrated that excessive APC were observed in participants with higher levels of N-terminal prohormone B-type natriuretic peptide[2,4]. These previous reports suggest that many general participants may have subclinical heart failure status, and APC are likely to become apparent with increased filling pressure. The adverse impact of APC on long-term outcome was apparent in participants with hypertension, which is associated with an increased LV filling pressure and diastolic dysfunction[23].

The other likely explanation is that APC triggers atrial fibrillation (AF) or is a forerunner of AF, resulting in the exacerbation of heart failure. Recent studies using 24-hour ECG monitoring demonstrated that frequent APC were correlated with a new occurrence of AF[4,6], while studies conducted in patients with acute stroke revealed that frequent APC predicted paroxysmal AF[20,24]. These findings suggest that APC could be a surrogate marker or a prodromal stage of AF, which is one of the independent prognostic factors in participants with heart failure. Furthermore, current concepts support the view that this paroxysmal or subclinical AF is also associated with an increased risk of acute stroke[25]. In our study, the presence of APC was associated with an increased risk of CVD mortality, particularly due to heart failure and stroke, reinforcing the possibility of an association between the presence of APC and the occurrence of AF, eventually leading to cardioembolic strokes[26].

Although in the present study, we have shown that CVD deaths occur twice as frequently in participants with APC than in those without APC, a previous large-scale study from the atherosclerosis risk in communities (ARIC) study reported conflicting results[3]. The latter study, however, focused only on sudden cardiac death or coronary heart disease, whereas the incidence of all CVD deaths, including heart failure or stroke—which are considered to be the most important outcomes related to APC—was not evaluated. These differences regarding endpoints might have reduced the prognostic impact of APC in that study.

It remains controversial whether an ECG examination should be performed in asymptomatic individuals. Our findings regarding APC are in concordance with western guidelines, which recommend the use of ECG screening in participants who have a high risk for CVD, such as patients with hypertension[27,28]. Screening for APC and other electrocardiographic abnormalities may be of assistance in performing further risk stratification in these patients; patients in a higher risk category may benefit from aggressive lifestyle modification or cardiovascular investigation. On the other hand, our results also demonstrated few associations of APC and CVD mortality in participants with lower risk for underlying heart diseases, especially in participants without hypertension, which may justify not performing ECG screening for apparently healthy population.

### Limitations

Our study has several limitations. First, although the participants in our cohort were limited to Japanese populations only, previous studies have demonstrated that the prognostic values of other ECG findings, proven in the United States or Europe, were also applicable to Japanese populations[8,12,17,29]. Asians reportedly have a different risk profile from western populations, with a significantly better overall cardiovascular prognosis[30,31], but a higher incidence of stroke than of coronary artery disease[32]. As shown in this study, the importance of APC as an independent predictor of stroke is beginning to be understood, and it is meaningful to evaluate the association of APC and adverse CVD outcomes, including stroke, in such a Japanese population. Second, sub-classifications of stroke events were not available. Although the etiology of stroke associated with premature complexes is likely to include the increased risk of cardioembolism, further studies are needed to confirm this. Last, we used a single ECG at baseline. Single biological measurements are known to be subject to variability. Therefore, the sensitivity of a single 12-lead ECG for the detection of premature complexes should be lower than that for more sensitive strategies, including 2-minute rhythm strips or a 24-hour ambulatory ECG. However, the presence of premature complexes on a single 12-lead ECG recorded in only 6 seconds might indicate that participants with such APC were likely to have frequent ectopic beats as a whole. 

## Conclusions

APC recorded during the screening ECG serve as an independent predictor of CVD deaths in a Japanese community-dwelling population. This tendency seemed to be most apparent in hypertensive participants, in whom the presence of APC was a predictor of CVD death, particularly due to heart failure and stroke.
